# Preoperative Nomogram for Predicting Sentinel Lymph Node Metastasis Risk in Breast Cancer: A Potential Application on Omitting Sentinel Lymph Node Biopsy

**DOI:** 10.3389/fonc.2021.665240

**Published:** 2021-04-26

**Authors:** Xi’E Hu, Jingyi Xue, Shujia Peng, Ping Yang, Zhenyu Yang, Lin Yang, Yanming Dong, Lijuan Yuan, Ting Wang, Guoqiang Bao

**Affiliations:** ^1^ Department of General Surgery, The Second Affiliated Hospital of Air Force Medical University, Xi’an, China; ^2^ The Second Clinical Medical College, Shaanxi University of Chinese Medicine, Xianyang, China; ^3^ Department of Vascular Surgery and Thyroid-Breast Surgery, The First Affiliated Hospital of Air Force Medical University, Xi’an, China

**Keywords:** breast cancer, nomogram, SLN, metastasis, ultrasound, external validation

## Abstract

**Background:**

Sentinel lymph node (SLN) biopsy is feasible for breast cancer (BC) patients with clinically negative axillary lymph nodes; however, complications develop in some patients after surgery, although SLN metastasis is rarely found. Previous predictive models contained parameters that relied on postoperative data, thus limiting their application in the preoperative setting. Therefore, it is necessary to find a new model for preoperative risk prediction for SLN metastasis to help clinicians facilitate individualized clinical decisions.

**Materials and Methods:**

BC patients who underwent SLN biopsy in two different institutions were included in the training and validation cohorts. Demographic characteristics, preoperative tumor pathological features, and ultrasound findings were evaluated. Multivariate logistic regression was used to develop the nomogram. The discrimination, accuracy, and clinical usefulness of the nomogram were assessed using Harrell’s C-statistic and ROC analysis, the calibration curve, and the decision curve analysis, respectively.

**Results:**

A total of 624 patients who met the inclusion criteria were enrolled, including 444 in the training cohort and 180 in the validation cohort. Young age, high BMI, high Ki67, large tumor size, indistinct tumor margins, calcifications, and an aspect ratio ≥1 were independent predictive factors for SLN metastasis of BC. Incorporating these parameters, the nomogram achieved a robust predictive performance with a C-index and accuracy of 0.92 and 0.85, and 0.82 and 0.80 in the training and validation cohorts, respectively. The calibration curves also fit well, and the decision curve analysis revealed that the nomogram was clinically useful.

**Conclusions:**

We established a nomogram to preoperatively predict the risk of SLN metastasis in BC patients, providing a non-invasive approach in clinical practice and serving as a potential tool to identify BC patients who may omit unnecessary SLN biopsy.

## Introduction

Breast cancer (BC) is the most frequently diagnosed malignant tumor among women worldwide. There were approximately 2.1 million new cases of BC worldwide in 2018, and 627 000 mortalities, seriously threatening women’s life and health (1). The presence of lymph node (LN) metastasis is one of the most important prognostic factors in BC patients; thus, the intervention on axillary lymph nodes (ALNs) has been the focus in the field of surgical treatment of BC (2). Identified as the first station of LN metastasis in BC, sentinel lymph nodes (SLNs) play a significant role in breast tumor invasion (3). SLN biopsy (SLNB) is a standard method for determining the metastatic status of ALN and assists clinicians in developing individualized treatment regimens. However, SLNB is not a completely benign procedure, as it is invasive and carries a risk of long-term comorbidities, such as sensory neuropathy, lymphedema, motor neuropathy, and pain (4, 5). In addition, it was reported that the SLN metastasis rate was 28.9-42.0% in clinically LN-negative BC patients, indicating that nearly half of these patients do not need SLNB (6). Therefore, an appropriate predictive nomogram is required to distinguish BC patients with a lower risk of SLN metastases from those at higher risk preoperatively to help doctors determine whether their patients could avoid SLNB.

Several previous studies have reported various risk factors associated with LN metastasis of BC, such as histological grade, lymphovascular invasion (LVI), and molecular indexes (7–10); however, whether they are sufficiently accurate to determine SLNB omission remains uncertain. Moreover, these predictive models are based on postoperative histopathological findings, which restrict their potential for non-invasive or preoperative applications. Thus, the development of a nomogram for preoperative use can help clinicians make more individualized clinical decisions.

Ultrasonography is a traditional medical imaging method that plays a significant role in BC detection, image-guided biopsy, and LN diagnosis (11). It has apparent advantages in breast assessments (12). It is a non-invasive diagnostic tool that is convenient, radiation-free, inexpensive, reusable, and has great potential for accurately evaluating the size and location of tumors, delineating the internal structure of LNs, and even diagnosing early metastatic lesions. Therefore, ultrasound imaging could provide a promising approach for predicting SLN metastasis in patients with BC.

Hence, this study aimed to establish a nomogram that combines clinicopathological characteristics and ultrasound findings to predict the SLN-metastasis risk of BC patients in a preoperative setting. We hope to explore a robust tool to help make a more favorable diagnosis of SLN in BC patients and contribute to assisting clinicians in selecting those who have the opportunity to avoid unnecessary SLNB, thereby allowing more individualized treatment for BC patients.

## Materials and Methods

### Patients

Female patients with pathologically confirmed BC, clinically negative LN metastasis, and who underwent SLNB were retrospectively included in this study. The training cohort comprised of BC patients from Xijing Hospital (the First Affiliated Hospital of Air Force Medical University) from January 1^st^, 2016, to January 1^st^, 2019 and BC patients from Tangdu Hospital (the Second Affiliated Hospital of Air Force Medical University) between January 1^st^, 2017, and January 1^st^, 2018. Clinicopathological data were obtained from medical records of the institutional database, and the ultrasound findings were collected from the Picture Archiving and Communication Systems (PACS), which is a database for medical images.

The inclusion criteria were as follows: (1) female patients with breast tumors diagnosed for the first time by pathological or clinical examinations; (2) had clinically negative LN metastasis (detected by medical imaging examination or puncture pathology); (3) underwent SLNB surgery in Xijing or Tangdu Hospital; and (4) had complete clinicopathological data, including breast and ALN ultrasound findings. Patients who were male, had distant metastases, bilateral lesions, or had previously received neoadjuvant therapies or breast surgeries were excluded. Ethical approval for this retrospective study was obtained (K202101-06), and informed consent was waived.

### Ultrasonography and Image Analysis

Ultrasound detection was performed in all patients using the Acuson S2000 system (Siemens Medical Solutions, Mountain View, CA, USA) with a transducer frequency of 5-12 MHz. In order for each quadrant of the breast to be examined thoroughly, the patients were kept in the supine, left-lateral, and right-lateral positions. Two sonographers with more than 5 years of experience in breast ultrasound examined the breast and ALNs using two-dimensional images and color Doppler spectra features. On the condition that the sonographers disagreed in assessing any parameters, the third sonographer with a 10-year experience of breast ultrasound would review the images and make the final decision. All the confirmed information, including both ultrasound images and reporting descriptions, was stored in the PACS database. The specific ultrasound characteristics collected were tumor size, tumor shape (regular or irregular, such as microlobulated, angular, or spiculated), tumor margin (distinct or indistinct), color Doppler flow (rich or poor), aspect ratio (<1 or ≥1; when under the ultrasound probe, the diameter of the tumor that is parallel to the skin is the horizontal line, and the diameter perpendicular to the skin is the vertical line; the aspect ratio refers to the ratio of the vertical line to the horizontal line of the tumor, which on the ultrasonic images is the ratio of the width of the tumor to its height), calcification (present or absent), whether ALNs are visible or enlarged, and Breast Imaging Reporting and Data System (BI-RADS) grade. All breast and LN information on B-mode and color Doppler flow were extracted and collected from the PACS. Upon extraction of the data from the PACS database, we assigned three breast ultrasound specialists to review and confirm the ultrasound images again, and then recorded the report results based on the ultrasound lexicon of the BI-RADS 5^th^ edition (13) and the color Doppler flow grading methods of Adler et al. (14) to avoid the subjectivity of the ultrasound findings in different centers as much as possible.

### Preoperative Pathologic and Immunohistochemical Analyses

The pathologic type, histologic grade, and status of ALN metastases were confirmed. The estrogen receptor (ER), progesterone receptor (PR), human epidermal growth factor receptor 2 (HER2), and Ki-67 status were evaluated by immunohistochemistry (IHC) in a preoperative pathological puncture. The cutoff point for ER- and PR-positive expression levels was 1% based on IHC results (15). HER2 positivity was defined as IHC staining of 3+ or fluorescence *in situ* hybridization (ISH) proliferation greater than 2 (15). The detailed ISH detection criteria for HER2 can be found in the NCCN guidelines (16). Ki67 scores were evaluated using the percentage of tumor cell nuclei with positive immunostaining above background, with greater than 30% showing elevated expression (17). BC molecular subtypes were categorized as luminal A, luminal B, HER2 amplified type and hormone receptor- (HR-) negative, HER2 amplified type and HR-positive, and triple-negative type according to the status of ER, PR, HER2, and Ki-67.

### SLNB and LN Histopathology

The patient was anesthetized and subsequently injected with 0.5 mL of 1% methylene blue or nano-carbon into the subcutaneous tissue at 3 o’clock, 6 o’clock, 9 o’clock, and 12 o’clock of the edge of the areola of the patient, followed by a light massage for 5 minutes. A radial incision was made at the lateral border of the pectoralis major muscle. The skin, subcutaneous, and adipose tissues were cut layer by layer. We separated and traced the lymphatic vessels, and the blue- and black-stained LNs were regarded as SLNs. The enlarged LNs palpated intraoperatively were also removed as SLNs. All SLNs were sent for rapid frozen pathological detection during the operation. If any of them were found to be positive for metastasis, further axillary lymph node dissection would be subsequently performed.

According to the AJCC 8th edition BC staging criteria (18), our main evaluation criteria for the final status of LN metastases were based on the postoperative pathologic diagnosis (pN). Negative LNs (pN0) were defined as no tumor cells or only isolated tumor cells that could be seen in histopathology (the maximum diameter of metastasis foci was less than 0.2 mm and the number of tumor cells in one section was less than 200), and positive LNs (pN[+]) were defined as the presence of macro-metastasis (maximum diameter of metastasis foci >2.0 mm) and micro-metastasis (maximum diameter of metastasis foci was 0.2-2.0 mm) of isolated tumor cells.

### Statistical Analyses

Descriptive statistics were used to delineate the clinicopathological characteristics of the study population. Continuous variables were presented as mean ± standard deviation (SD) or median and interquartile range and were compared using an unpaired two-independent-simple Student’s t-test or Mann-Whitney U test. Categorical variables were expressed as counts and percentages and compared using the Chi-square test or Fisher’s exact test.

Univariate logistic regression analysis was used to select the candidate variables of the training cohort, and variables with a p-value <0.2 were included in the multivariable regression model as independent predictive factors associated with SLN metastasis of BC (19). Backward stepwise selection was applied using the likelihood ratio test with Akaike’s information criterion (AIC) as the stopping rule. To provide the clinician with a quantitative tool to predict the individual probability of SLN metastasis, we established a nomogram based on the multivariate logistic regression analysis in the training cohort, using the rms package of R (R Project for Statistical Computing, RRID: SCR_001905; version 4.0.3; http://www.r-project.org). The predictive performance was measured by both internal and external validation by plotting the calibration curve of 1000 bootstrap samples and calculating the concordance index (C-index) to reduce the overfitting bias. Sensitivity, specificity, predictive value, and likelihood ratios with their 95% confidence intervals (CIs) were evaluated to assess the accuracy of the model using the receiver operation characteristic curve (ROC) analysis. Decision curve analysis (DCA) was conducted to estimate the potential clinical usefulness of the nomogram by quantitative analysis of the net benefits at different threshold probabilities (20). Our research data were processed in Stata (Stata, RRID: SCR_012763) version 15.0 for Windows (StataCorp, Texas, USA) and R version 4.0.3. Statistical significance was defined as a two-sided P value <.05.

## Results

### Clinicopathologic Characteristics

**Figure 1:**
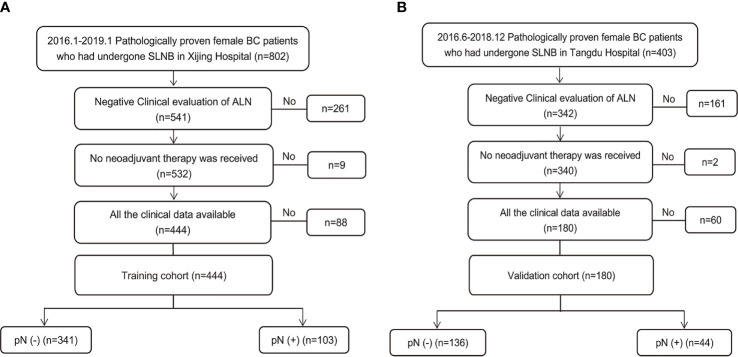
Study population enrolment in the training and validation cohort. **(A)** Study population enrolment in the training cohort; **(B)** Study population enrolment in the validation cohort. SLNB, sentinel lymph node biopsy; BC, breast cancer; pN(-), negative lymph node metastasis confirmed by pathology; pN(+), positive lymph node metastasis confirmed by pathology.

During the study period, 1205 consecutive patients diagnosed with BC based on preoperative pathology underwent SLNB. Of these, 624 patients who met the inclusion criteria were enrolled (**Figure 1**). The training set consisted of 444 patients from Xijing Hospital (positive vs. negative SLN metastasis: 103 vs. 341), and the validation set included 180 patients from Tangdu Hospital (positive vs. negative SLN metastasis: 44 vs. 136). The sample size of this study met the standard of 10 outcome events per predictor variable (EPV) (21, 22). The comparison between patients with positive and negative final SLN status showed statistically significant differences in BMI, overall TNM stage, clinical T classification, tumor size, presence of tumor calcification, and aspect ratio of the tumor (**Table S1**).

Patients’ baseline characteristics in the training (mean age, 51.19 ± 0.52 years; range, 23 to 80 years) and validation cohort (mean age, 51.31 ± 0.82 years; range, 23 to 82 years) are given in **Table 1**. The positive rate of SLN metastasis was 23.2% and 24.4%, respectively, in these two cohorts, with no significant differences in SLN prevalence (P=0.74). There were also no significant differences in age, BMI, menstrual status, lesion position, LN stage, histological type, ER status, PR status, Ki67, BI-RADS, tumor shape, and color Doppler flow between the training and validation cohorts; however, differences in some clinicopathologic characteristics were observed in patients of these two cohorts owing to the spatial span of the different institutions, according to our study. In terms of clinicopathological features, the tumor stage and pathological stage were lower in the training set than in the validation set. Based on the pathological evaluation, approximately two-thirds of the patients in the training cohort were in stage T1 (62.4%), but in the validation cohort were in stage T2 (65.6%). More patients in the training set had primary tumors at histological grade 1 than in the validation set (21.1% vs. 6.1%; P<.001).

**Table 1:** Participant baseline characteristics in two cohorts.

Characteristics	Training data (n=444) (%)	Validation data (n=180) (%)	P Value
**Age, media (IQR), years**	49.0 (44.0, 58.5)	49.5 (44.0, 58.5)	0.90
**BMI, media (IQR), kg/m^2^**	23.3 (21.6, 24.8)	23.4 (21.5, 25.4)	0.06
**Menstrual status**			0.13
Pre-	237 (53.4)	84 (46.7)	
Post-	207 (46.6)	96 (53.3)	
**Lesion position**			0.89
OUQ	255 (57.4)	109 (60.6)	
OLQ	69 (15.5)	26 (14.4)	
IUQ	83 (18.7)	29 (16.1)	
ILQ	26 (5.9)	10 (5.6)	
Center	11 (2.5)	6 (3.3)	
**T classification**			**<0.001**
Tis	67 (15.1)	1 (0.6)	
T1	277 (62.4)	59 (32.8)	
T2	100 (22.5)	118 (65.6)	
T3	0 (0.0)	2 (1.1)	
**N classification**			0.21
N0	344 (77.5)	136 (75.6)	
N1	97 (21.8)	39 (21.7)	
N2	2 (0.5)	3 (1.7)	
N3	1 (0.2)	2 (1.1)	
**Overall TNM stage**			**<0.001**
Ia	246 (55.4)	48 (26.7)	
Ib	68 (15.3)	11 (6.1)	
IIa	71 (16.0)	86 (47.8)	
IIb	29 (6.5)	28 (15.6)	
III	2 (0.5)	6 (3.3)	
**Histological type**			0.96
Ductal	353 (79.5)	144 (80.0)	
Lobular	35 (7.9)	13 (7.2)	
Others	56 (12.6)	23 (12.8)	
**Histological grade**			**<0.001**
I	94 (21.1)	11 (6.1)	
II	314 (70.0)	153 (85.0)	
III	36 (8.1)	16 (8.9)	
**Subtype**			**0.001**
Luminal A	264 (59.5)	92 (51.1)	
Luminal B	77 (17.3)	23 (12.8)	
HER2+ (HR-)	29 (6.5)	14 (7.8)	
HER2+ (HR+)	25 (5.6)	27 (15.0)	
TNBC	49 (11.0)	24 (13.3)	
**ER**			0.23
Negative	80 (18.8)	40 (22.2)	
Positive	364 (82.0)	140 (77.8)	
**PR**			0.53
Negative	122 (27.5)	45 (25.0)	
Positive	322 (72.5)	135 (75)	
**HER2**			**0.001**
Negative	390 (87.8)	139 (77.2)	
Positive	54 (12.2)	41 (22.8)	
**Ki67, media (IQR), %**	18 (10, 30)	20 (10, 30)	0.17
**US Findings**			
**BI-RADS**			0.70
4A	65 (14.6)	23 (12.8)	
4B	74 (16.7)	35 (19.4)	
4C	112 (25.2)	52 (28.9)	
5	87 (19.6)	32 (17.8)	
6	106 (23.9)	38 (21.1)	
**Multifocality**			**0.01**
yes	96 (21.6)	23 (12.8)	
no	348 (78.4)	157 (87.2)	
**Tumor size,** **media (IQR), cm**	1.6 (1.2, 2.1)	2.1 (1.7, 2.7)	**<0.001**
**Tumor shape**			0.44
regular	27 (6.1)	14 (7.8)	
irregular	417 (93.9)	166 (92.2)	
**Tumor Margin**			**<0.001**
distinct	46 (10.4)	52 (28.9)	
indistinct	398 (89.6)	128 (71.1)	
**Inner echo**			**0.03**
even	72 (16.2)	17 (9.4)	
uneven	372 (83.8)	163 (90.6)	
**Calcification**			**<0.001**
present	209 (47.1)	52 (28.9)	
absent	235 (52.9)	128 (71.1)	
**Color Doppler flow**			0.18
rich	424 (95.5)	176 (97.8)	
poor	20 (4.5)	4 (2.2)	
**Aspect ratio**			**<0.001**
≥1	34 (7.7)	66 (36.7)	
<1	410 (92.3)	114 (63.3)	

OUQ, outer upper quadrant; OLQ, outer lower quadrant; IUQ, inner upper quadrant; ILQ, inner lower quadrant; HR, hormone receptor; TNBC, triple-negative breast cancer; ER, estrogen receptor; PR, progesterone receptor; US, ultrasound.

Bold value indicates statistical significance.

Moreover, more patients were diagnosed with the HER2 positive subtype in the validation cohort than in the training cohort (22.8% vs. 12.1%; P=.001). Similarly, according to the preoperative ultrasound examination results, both training and validation cohorts had more patients with a single lesion, but fewer patients with multifocal BC tumors in the former (P=0.01). In addition, compared with the patients in the validation set, the primary tumor in the training set was generally more extensive, with indistinct margins, more uneven internal echoes, more calcifications, and more lesions with an aspect ratio <1 (P<.001, <.001, <0.05, =.001, and =.001, respectively). The variety in these different baseline characteristics in the two cohorts may be caused by differences in the studied population from the two centers after excluding those who received neoadjuvant therapy. These differences can better indicate the generalizability and predictive capacity of the model application.

### Nomogram Development of SLN-Metastasis Risk

#### Univariate Logistic Analysis and Candidate Factors Selection

All variables incorporated in the model were based on the data obtained preoperatively; therefore, postoperative indicators such as pathological T and N classifications, pathological TNM stage, and histological grade were not included. The results of the univariate logistic analysis are presented in **Table 2**. Variables with p-values <0.2 were age (P=0.04), BMI (P=0.02), PR status (P=0.15), Ki67 (P=0.04), tumor size (P<0.001), inner echo of tumor (P=0.09), tumor calcification (P<0.001), color Doppler flow (P=0.17), and aspect ratio (P<0.001). These variables were included in the multivariable regression model as the candidate predictive factors associated with SLN metastasis risk.

**Table 2:** Univariate Logistic Regression Analysis of SLN Metastasis Based on Preoperative Data in the Training Cohort.

Variables	P Value	OR (95% CI)
**Age (years)**	**0.04**	0.98 (0.96-1.00)
**BMI (kg/m^2^)**	**0.02**	1.12 (1.02-1.22)
**Menstrual status (post- vs. pre-)**	0.50	0.86 (0.55-1.34)
**Lesion position**		
OLQ vs. OUQ	0.62	0.88 (0.53-1.46)
IUQ vs. OUQ	0.41	0.82 (0.51-1.32)
ILQ vs. OUQ	0.79	0.90 (0.42-1.93)
Center vs. OUQ	0.64	1.28 (0.46-3.54)
**Histological type**		
lobular vs. ductal	0.78	0.91 (0.47-1.77)
others vs. ductal	0.98	1.01 (0.60-1.70)
**Subtype**		
Luminal B vs. Luminal A	0.56	0.86 (0.51-1.45)
HER2+ (HR-) vs. Luminal A	0.35	1.39 (0.70-2.74)
HER2+ (HR+) vs. Luminal A	**<0.001**	3.10 (1.71-5.61)
TNBC vs. Luminal A	0.22	1.41 (0.82-2.42)
**ER (negative vs. positive)**	0.32	0.76 (0.44-1.31)
**PR (negative vs. positive)**	**0.15**	0.71 (0.44-1.14)
**HER2 (negative vs. positive)**	**0.06**	1.79 (0.97-3.32)
**Ki67**	**0.04**	1.01 (1.00-1.02)
**US Findings**		
** Multifocality (multiple vs. single)**	0.25	0.71 (0.41-1.26)
** Tumor size (cm)**	**<0.001**	3.33 (2.42-4.59)
** Tumor shape (irregular vs. regular)**	0.73	0.85 (0.35-2.08)
** Margin (indistinct vs. distinct)**	0.22	0.66 (0.34-1.29)
** Inner echo (uneven vs. even)**	**0.09**	1.82 (0.92-3.61)
** Calcification (present vs. absent)**	**<0.001**	8.97 (5.10-15.78)
** Color Doppler flow (rich vs. poor)**	**0.17**	2.81 (0.64-12.34)
** Aspect ratio (<1 vs. ≥1)**	**<0.001**	0.10 (0.05-0.22)

CI, confidence interval; OUQ, outer upper quadrant; OLQ, outer lower quadrant; IUQ, inner upper quadrant; ILQ, inner lower quadrant; HR, hormone receptor; TNBC, triple-negative breast cancer; ER, estrogen receptor; PR, progesterone receptor; US, ultrasound.

Bold value are variables with P <0.2 which are candidate variables in multivariable regression analysis.

#### Multivariate Logistic Analysis Nomogram Development

**Figure 2:**
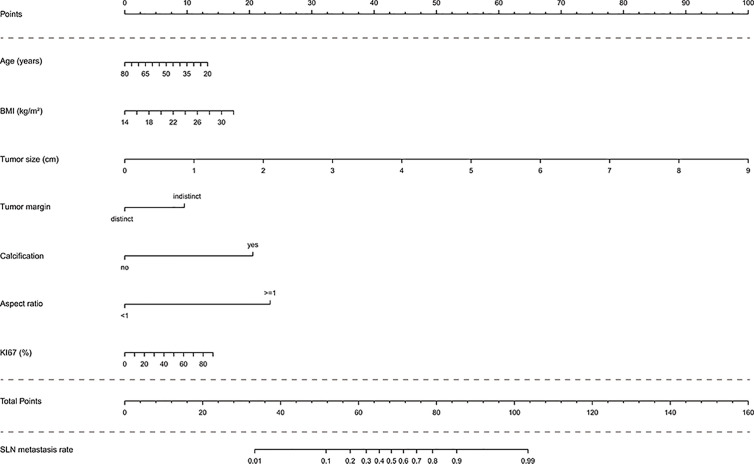
Nomogram to predict the rate of SLN metastasis in clinically LN-negative breast cancer patients. The nomogram to predict SLN-metastasis-risk was created based on the above seven predictive factors. To use the nomogram, the value of each patient is placed on each variable axis and a line is drawn upward to determine the number of received points for each variable value. The sum of these numbers is located on the total point axis and a line down the bottom axis is drawn to determine the probability of SLN metastasis.

In the multivariate analysis, with results reported as odds ratio (95% CI), young age (0.97 [0.94-1.00]), high BMI (1.14 [0.99-1.31]), high Ki67 (1.02 [1.00-1.04]), large tumor size (4.29 [2.88-6.39]), indistinct tumor margins (0.29 [0.10-0.79]), calcified tumor (14.79 [6.45-33.94]), and an aspect ratio ≥1 (0.05 [0.02-0.13]) were independent predictive factors associated with the risk of SLN metastasis (**Table 3**). These independent predictors were used to form the SLN metastasis risk estimation nomogram, as shown in **Figure 2**.

**Table 3:** Multivariate logistic regression analysis of SLN metastasis based on preoperative data in the training cohort.

Variables	β^#^	P value	OR (95% CI)
**Age (per 1-year increase)**	-0.03	0.059	0.97 (0.94-1.00)
**BMI (per 0.1kg/m^2^ increase)**	0.14	0.071	1.14 (0.99-1.31)
**Ki67 (per 1% increase)**	0.02	0.016	1.02 (1.00-1.04)
Tumor size^*^ (per 0.1cm increase)	1.46	<.001	4.29 (2.88-6.39)
Tumor margin^*^ (distinct vs. indistinct)	-1.25	0.015	0.29 (0.10-0.79)
Calcification^*^ (present vs. absent)	2.69	<.001	14.79 (6.45-33.94)
Aspect ratio^*^ (<1 vs. ≥1)	-3.06	<.001	0.05 (0.02-0.13)
**Constant**	0.19	0.937	1.20 (0.01-107.65)

^#^Unstandardized β coefficients were calculated from the multivariate logistic regression analysis based on stepwise regression (AIC: 267.85).

^*^Variables based on US results.

CI, confidence interval.

### Nomogram Validation of SLN-Metastasis Risk

#### Calibration of the Nomogram

**Figure 3:**
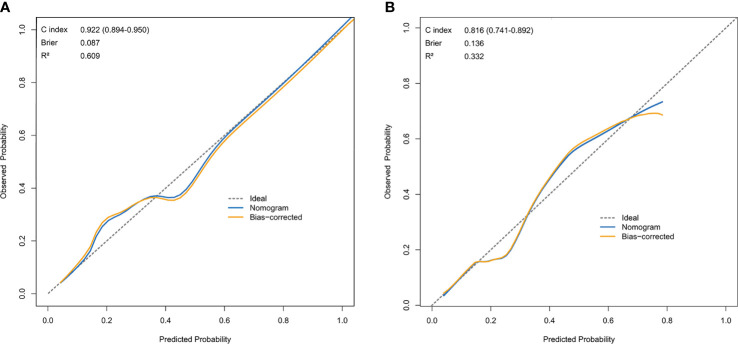
Calibration curve comparing predicted and actual SLN-metastasis-risk probabilities. **(A)** Calibration curve of the nomogram in the training cohort. **(B)** Calibration curve of the nomogram in the validation cohort. The calibration curve describes the calibration of the model according to the consistency between the predicted risk of SLN metastasis and the observed results of SLN metastasis. The x-axis represents the predicted probability of SLN metastasis. T The y-axis represents the actual SLN metastasis probability. he gray dotted line represents the perfect prediction of the ideal model. The solid blue line represents the prediction of the nomogram, and the solid orange line represents the bootstrap-corrected estimates. A well calibrated curve of a nomogram would be near the ideal line.

The resulting model was validated both internally and externally using bootstrap validation. The nomogram demonstrated good accuracy in estimating the risk of SLN metastasis with a C-index of 0.92 (95% CI, 0.89-0.95). In addition, calibration plots graphically showed good agreement on the presence of SLN metastasis between the risk estimation by the nomogram and histopathologic confirmation on surgical specimens (**Figure 3A**). In the validation cohort, the nomogram displayed a C index of 0.82 (95% CI, 0.74-0.89) to estimate SLN metastasis risk. There was also a good calibration curve for risk estimation (**Figure 3B**).

#### Accuracy of the Nomogram

**Figure 4:**
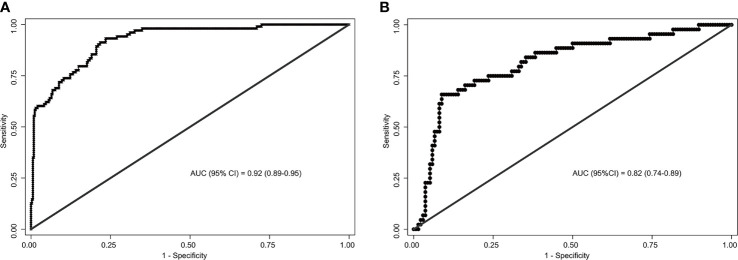
Receiver operating characteristics (ROC) curves of the nomograms in training and validation cohort. **(A)** The ROC curve of the training cohort; **(B)** The ROC curve of the validation cohort. The nomogram had a good discriminative performance with Area under ROC curve (AUC) (95% confidence interval) of 0.92 (95% CI: 0.89-0.95) and 0.82 (95% CI: 0.74-0.89) in the training and validation cohort, respectively.

The area under the ROC curve (AUC) for internal and external validation was 0.92 (**Figure 4A**) and 0.82 (**Figure 4B**), respectively. The cutoff score was 55 when the Youden index was at the maximum. Patients with a score of 55 or more were at a high risk of SLN metastasis. Using 55 as a cutoff score, the sensitivity, specificity, accuracy, positive predictive value, and negative predictive value were 66%, 91%, 85%, 71%, and 89% in the training cohort and 93%, 77%, 80%, 55%, and 97% in the validation cohort, respectively (**Table 4**).

**Table 4:** Accuracy of the prediction score of the nomogram for estimating the risk of SLN metastasis.

Variables	P Value (95% CI)	
	Training Cohort	Validation Cohort
**AUC/C-Index**	0.92 (0.89-0.95)	0.82 (0.74-0.89)
**Cutoff score**	55	55
**Sensitivity**	0.66 (0.50-0.79)	0.93 (0.86-0.97)
**Specificity**	0.91 (0.85-0.95)	0.77 (0.72-0.81)
**Accuracy**	0.85 (0.79-0.90)	0.80 (0.76-0.84)
**Positive predictive value**	0.71 (0.54-0.83)	0.55 (0.47-0.62)
**Negative predictive value**	0.89 (0.83-0.94)	0.97 (0.94-0.99)

CI, confidence interval; AUC, the area under the receiver operating curve; C-Index, concordance index.

#### Clinical Usefulness Evaluation of the Nomogram

**Figure 5:**
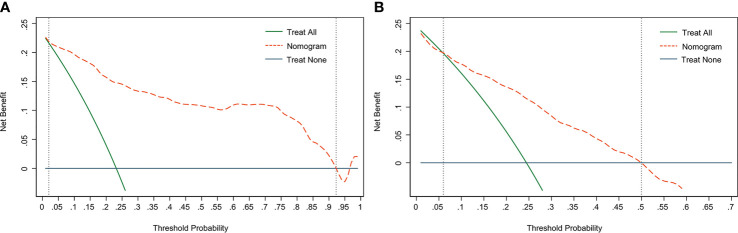
Decision curve analysis (DCA) of the nomogram. **(A)** The DCA curve of the training cohort; **(B)** The DCA curve of the validation cohort. The orange line shows the nomogram. The green line represents the assumption that all patients have undergone SLNB. The dark blue line represents the assumption that no patients have undergone SLNB. The decision curve revealed that it was more benefit to use the nomogram in our study to predict SLN metastasis than the treat-all-patients scheme or the treat-none scheme, when the threshold probability of a patient is 2%-92%, and 6%-50% in the training cohort and validation cohort, respectively.

DCA is used to assess the benefits of diagnostic models covering a range of patient preferences for the risks of under- and overtreatment to facilitate more reasonable decisions regarding the model selection and use (23). The net benefit in DCA was calculated by subtracting the proportion of all false-positive patients from the ratio of true positives and weighing the relative harm of abandoning treatment and the adverse outcomes of unnecessary treatment. The DCA in the current study showed that the nomogram of the SLN metastasis model used in our study was more effective than all-patient treatment or no treatment if the threshold probability ranged from 2% to 92% in the training cohort (**Figure 5A**), and from 6% to 50% in the validation cohort (**Figure 5B**).

## Discussion

Among the currently available prediction tools, the nomogram has high accuracy and good discriminability, as well as convenient and important in clinical use (24), which can change the treatment pattern of BC patients (25). Previous nomograms of breast tumors mainly focused on the risk of non-SLN metastasis (26–28) or total LN metastasis (10, 29–31) to predict the possibility of axillary lymph node dissection omission to appropriately minimize the scope of axillary surgery. However, few studies have focused on the omission of SLNB. Additionally, most of the parameters included in these models were pathological indicators obtained postoperatively, such as tumor molecular subtypes, tumor grade, and LVI, which were difficult to accurately obtain before surgery, thus restricting their usage in the preoperative setting. In addition, some studies have attempted to introduce radiomics examinations and pathological indicators to predict breast malignancy or LN metastasis (32–35), but further clinical validation is required.

In this study, we included two cohorts of BC patients from different centers to develop and validate a predictive nomogram of SLN-metastasis risk, combining various indicators that were easily available preoperatively, including clinicopathological characteristics and detailed ultrasound diagnostic results. Seven preoperative parameters were identified as independent predictive risk factors. Although the spatial disconnection existed between the two study cohorts, the nomogram achieved a robust predictive performance with a C-index and accuracy of 0.92, 0.85, 0.82, and 0.80 in the training and validation cohorts, respectively. In addition, the calibration curves also fit well. For clinical use of the nomogram, we adopted 55 as the cutoff value and summarized the sensitivity, specificity, accuracy, positive predictive value, and negative predictive value to evaluate the quality of the model. The nomogram might enable 91% and 77% (**Table 4**) of patients in the training and validation sets, respectively, in our study, to avoid unnecessary SLNB. It provides a new method for preoperative and non-invasive prediction of SLN metastasis in BC patients, which has a potential predictive reference value for the omission of SLNB in the clinic.

Currently, two ongoing clinical trials, the SOUND trial (ClinicalTrials.gov identifier NCT02167490) (36) and the NAUTILUS trial (ClinicalTrials.gov identifier NCT04303715), have some similarities to this study. A detailed comparison of the three studies is presented in **Table S2**. The aim of the three studies was similar, which was to establish a minimally invasive treatment omitting SLNB of BC; however, these two clinical trials have strict inclusion criteria. Among these criteria, tumor size is the only one that is associated with the risk of LN metastasis. However, this study was designed to build a prediction system in which a variety of indicators probably associated with lymph node metastasis were retrospectively analyzed, and seven of them were selected and developed a nomogram to predict the risk of SLN metastasis. Patients with a low risk evaluated by the model may omit SLNB.

In the LN metastasis predictive nomogram, young age, large tumor size, tumor calcifications, high BMI, and Ki67 status were associated with an increase in LN metastasis in BC (7, 10, 30, 31, 37–40). Likewise, our study showed that these factors were also related to an increased probability of SLN metastasis in BC. In addition, we found that an indistinct margin and the aspect ratio of the tumor based on ultrasonography were independent predictive factors for SLN metastasis.

Ultrasound imaging is a promising tool for predicting LN metastasis in patients with BC and is an important imaging method for preoperative BC screening and evaluation (34). In this study, the tumor size (the maximum diameter of the tumor) based on ultrasound imaging was the most significant predictive factor for SLN metastasis. This significantly contributed to the model with an OR of 4.29, which suggested that patients were 4.29 times more likely to have SLN metastasis with every 0.1 cm increase in tumor size. Tumor size is easily and quickly measured by ultrasonography, which enables the model to be more applicable in the clinic.

Calcification is a deposit of calcium in the breast that appears as white, opaque spots, and scattered or partial agglutination on ultrasound images. Currently, calcification in breast imaging is primarily used in the diagnosis of cancer, noting that calcification is associated with invasive BC or ductal carcinoma *in situ* (41). When the tumor is rapidly growing with an active metabolism, the lack of oxygen and nutrients results in ischemic necrosis and calcium deposition, leading to calcifications appearing on the ultrasound image (42). It has been reported that calcifications not only play a crucial role in BC diagnosis but also have prognostic value, due to their correlation with high histological grade (43, 44), LN-positive status (44), HR-negative status (45), and HER2-positive status (46). Similarly, the calcification in breast tumors was also found to be associated with SLN-metastasis according to our research, with a 13.79-fold increased risk compared with uncalcified BC lesions, suggesting that clinicians should be alert about calcified breast tumors.

To the best of our knowledge, this is the first forecasting model consisting of tumor margin and aspect ratio for predicting SLN metastasis. Previous studies revealed that breast tumors with a non-circumscribed margin had a higher probability of LVI (47), noting that the indistinct margin of the tumor may provide important information regarding neoplasm invasion. According to our study, SLN-positive BC tumors were more likely to have an aspect ratio greater than 1. For patients with invasive BC, the tumor does not routinely grow in the plane but grows vertically or away from the horizontal direction, so the overall volume of the tumor will expand, resulting in a larger aspect ratio.

According to our study, younger patients were more likely to develop SLN metastases. Several studies have demonstrated that age at diagnosis is an independent prognostic factor in patients with metastatic BC (48–51). In most cases, breast tumors in younger women behave more aggressively than those in older women and have a higher rate of local recurrence (52, 53). The exact definition of young women in breast oncology settings varies, with most articles identifying women <35, 40, or 45 years as young (54). However, several studies support that premenopausal women with BC should be further subdivided into very early stages of disease (<40 years) and relatively early stages (40-49 years) (55). To discover a more subtle effect, we incorporated age as a continuous variable in our model. The result implied that for each 1-year younger age at BC diagnosis, the risk of SLN metastasis would increase by 3%.

High BMI is associated with tumor invasiveness, shorter disease recurrence, and more significant mortality in patients with BC. The POSH study following 2 956 young British patients from 2001 to 2007 reported a positive association between BMI and larger tumor size, higher tumor histological grade, and positive LN involvement (56). The American Cancer Society’s Cancer Prevention Study II, which followed 495 477 women from 1982 to 1998, reported a positive association between BMI and BC mortality: women with a BMI of 40 kg/m^2^ had more than a two-fold increased risk of death compared with those with a BMI of 18 to 24.9 kg/m^2^ (57). Moreover, a meta-analysis of 52 904 subjects showed that BMI increased the LN metastasis risk of BC, and for every 1 kg/m^2^ increment in BMI, the risk of LN metastasis increased by 0.89 (58). This may be attributed to the increase in pro-inflammatory cytokines in the local and circulation caused by the high BMI, which promotes tumor growth, angiogenesis, invasion, and metastasis (59). Our study also revealed that high BMI was more likely to cause SLN metastasis, and for each 0.1 kg/m^2^ increase in BMI, the risk of SLN metastasis increased by 14%, which indicated that losing weight and maintaining a healthy lifestyle intervention would be beneficial to BC patients.

As a biomarker of tumor proliferation, Ki67, which is a prognostic indicator that provides a rapid method to assess the proportion of proliferating cells in a tumor, and a higher level of Ki67 indicates more proliferating tumor cells (60). A previous study reported that BC patients with higher expression of Ki67 had significantly poorer 10-year disease-free survival than those with lower expression (61). In most studies concerning the association between Ki67 and metastasis of breast tumors, Ki67 was frequently treated as a classification variable; hence, the relationship between them could only be roughly estimated. In this study, we included Ki67 as a continuous variable, and the results showed that every 1% increase in the expression level of Ki67 increased the risk of SLN-metastasis by 1.02 times.

This study explored the probability of omitting SLNB from the perspective of retrospective observation and analysis. Based on the preoperative predictions, the nomogram may also serve as a useful tool to select BC patients for further randomized clinical trials of omission of SLNB. Additionally, the nomogram may also be used in clinical trials to evaluate the efficacy of breast-conserving surgery in patients with early BC and other subgroups with different risks of SLN metastasis.

However, this study has several limitations. First, this retrospective study excluded males, patients who had undergone neoadjuvant therapy, and those with incomplete clinical data; therefore, this model may not be applicable to them. Second, ultrasound findings, including tumor size, aspect ratio, and tumor margin, were assessed by the radiologist in a subjective manner. For the aspect ratio, a 0.1-difference in the measurement can add approximately 25 points. In addition, differences in the assessment of tumor margins can result in various tumor sizes. Compared to other objective factors (age, BMI, calcification), these subjective factors are the primary factors that may influence the results of the final nomogram, which could be a critical issue to consider when being applied in the clinic to evaluate patients who would potentially be able to omit SLNB. Third, although the nomogram achieved a favorable predictive performance, it still had a 34% and 7% false-negative-rate in the training and validation cohorts, respectively. Therefore, it is necessary to establish more accurate and uniform ultrasound assessment criteria for BC in the future, and prospective cohort studies in terms of SLNB omitting with more subgroups, larger samples, and more centers are still required.

## Conclusions

In summary, we established a nomogram combining clinicopathological characteristics and ultrasound features, including age, BMI, Ki67, tumor size, tumor margin, calcifications, and aspect ratio, to predict SLN-metastasis risk in BC patients before surgery. The nomogram provides a non-invasive approach in preoperative clinical decision-making and individualized treatment, which also has the potential to serve as a helpful and convenient tool to identify BC patients who have an opportunity to omit SLNB.

## Data Availability Statement

The raw data supporting the conclusions of this article will be made available by the authors, without undue reservation.

## Author Contributions

Xi’eH: data collection, data analysis and interpretation, and drafting the article. GB and TW: conception of the work, critical revision of the article and final approval of the version to be published. JX, SP, PY, ZY and LYa: data collection. LYu, JX and YD: critical revision of the article. All authors contributed to the article and approved the submitted version.

## Funding

This work was supported by the National Natural Science Foundation of China (NO: 81572916, 81502424).

## Conflict of Interest

The authors declare that the research was conducted in the absence of any commercial or financial relationships that could be construed as a potential conflict of interest.
