# Supplementary material for: Preoperative Nomogram for Predicting Sentinel Lymph Node Metastasis Risk in Breast Cancer: A Potential Application on Omitting Sentinel Lymph Node Biopsy
Source: Front Oncol. 2021 Apr 26;11:665240. doi: 10.3389/fonc.2021.665240 (PMC8107679; doi:10.3389/fonc.2021.665240)
Supplement: Supplementary file 1 [file DataSheet_1.pdf]

**Table S1 |** Clinicopathologic characteristics of the training and the validation cohort with or without SLN metastasis

| Clinicopathologic features | Training cohort (n=444) | | | Validation cohort (n=180) | | |
|---|---|---|---|---|---|---|
| | No SLN metastasis (n=341) (%) | SLN metastasis (n=103) (%) | P value | No SLN metastasis (n=136) (%) | SLN metastasis (n=44) (%) | P value |
| **Age (y)** | | | 0.094 | | | 0.094 |
| ≤35 | 17 (5.0) | 9 (8.7) | | 6 (4.4) | 6 (4.4) | |
| 36-55 | 208 (61.0) | 69 (67.0) | | 87(64.0) | 24 (54.5) | |
| >55 | 116 (34.0) | 25 (24.3) | | 43 (31.6) | 14 (31.8) | |
| **BMI (kg/m$^2$)** | | | **0.015** | | | 0.482 |
| <18.5 | 12 (3.5) | 2 (1.9) | | 1 (0.7) | 1 (2.3) | |
| 18.5-24.9 | 265 (77.7) | 68 (66.0) | | 91 (66.9) | 32 (72.7) | |
| ≥25 | 64 (18.8) | 33 (32.0) | | 44 (32.4) | 11 (25.0) | |
| **Menstrual status** | | | 0.496 | | | 0.391 |
| Pre- | 179 (52.5) | 58 (56.3) | | 61 (44.9) | 23 (52.3) | |
| Post- | 162 (47.5) | 45 (43.7) | | 75 (55.1) | 21 (47.7) | |
| **Lesion position** | | | 0.705 | | | 0.327 |
| OUQ | 198 (58.1) | 57 (55.3) | | 84 (61.8) | 25 (56.8) | |
| OLQ | 50 (14.7) | 18.4 (4.3) | | 17 (12.5) | 9 (20.5) | |
| IUQ | 66 (19.4) | 17 (16.5) | | 24 (17.6) | 5 (11.4) | |
| ILQ | 18 (5.3) | 8 (7.8) | | 8 (5.9) | 2 (4.5) | |
| Center | 9 (2.6) | 2 (1.9) | | 3 (2.2) | 3 (6.8) | |
| **Overall TNM stage** | | | **0.004** | | | **<0.001** |
| 0 | 25 (7.3) | 3 (2.9) | | 1 (0.7) | 0 (0.0) | |
| Ia | 197 (57.8) | 49 (47.6) | | 47 (34.6) | 2 (4.5) | |
| Ib | 53 (15.5) | 15 (14.6) | | 86 (63.2) | 9 (20.5) | |
| IIa | 49 (14.4) | 22 (21.4) | | 1 (0.7) | 25 (56.8) | |
| IIb | 15 (4.4) | 14 (13.6) | | 1 (0.7) | 5 (11.4) | |
| III | 2 (0.6) | 0 (0.0) | | 0 (0.0) | 3 (6.8) | |
| **Clinical T classification** | | | **0.003** | | | 0.464 |
| Tis | 53 (15.5) | 14 (13.6) | | 1 (0.7) | 0 (0.0) | |
| T1 | 224 (65.7) | 53 (51.5) | | 48 (35.3) | 11 (25.0) | |
| T2 | 64 (18.8) | 36 (23.2) | | 86 (63.2) | 32 (72.7) | |
| T3 | 0 (0.0) | 0 (0.0) | | 1 (0.7) | 1 (2.3) | |
| **Histological type** | | | 0.075 | | | 0.330 |
| Ductal | 279 (81.8) | 74 (71.8) | | 112 (82.4) | 32 (72.7) | |
| Lobular | 25 (7.3) | 10 (9.7) | | 8 (5.9) | 5 (11.4) | |
| Others | 37 (10.9) | 19 (18.4) | | 16 (11.8) | 7 (15.9) | |

| | | | | | | |
|---|---|---|---|---|---|---|
| **Histological grade** | | | 0.176 | | | 0.091 |
| I | 77 (22.6) | 17 (16.5) | | 6 (4.4) | 5 (11.4) | |
| II | 240 (70.4) | 74 (71.8) | | 120 (88.2) | 33 (75.0) | |
| III | 24 (7.0) | 12 (11.7) | | 10 (7.4) | 6 (13.6) | |
| **Subtype** | | | 0.298 | | | 0.362 |
| Luminal A | 203 (59.5) | 61 (59.2) | | 69 (50.7) | 23 (52.3) | |
| Luminal B | 64 (18.8) | 13 (12.6) | | 14 (10.3) | 9 (20.5) | |
| HER2+ (HR-) | 19 (5.6) | 10 (9.7) | | 12 (8.8) | 2 (4.5) | |
| HER2+ (HR+) | 17 (5.0) | 8 (7.8) | | 21 (15.4) | 6 (13.6) | |
| TNBC | 38 (11.1) | 11 (10.7) | | 20 (14.7) | 4 (9.1) | |
| **ER** | | | 0.314 | | | 0.115 |
| Negative | 58 (17.0) | 22 (21.4) | | 34 (25.0) | 6 (13.6) | |
| Positive | 283 (83.0) | 81 (78.6) | | 102 (75.0) | 38 (86.4) | |
| **PR** | | | 0.151 | | | 0.109 |
| Negative | 88 (25.8) | 34 (33.0) | | 38 (27.9) | 7 (15.9) | |
| Positive | 253 (74.2) | 69 (67.0) | | 98 (72.1) | 37 (84.1) | |
| **HER2** | | | 0.060 | | | 0.403 |
| Negative | 305 (89.4) | 85 (82.5) | | 103 (75.7) | 36 (81.8) | |
| Positive | 36 (10.6) | 18 (17.5) | | 33 (24.3) | 8 (18.2) | |
| **KI67** | | | 0.079 | | | 0.876 |
| <30% | 246 (72.1) | 65 (63.1) | | 91 (66.9) | 30 (68.2) | |
| >=30% | 95 (27.9) | 38 (36.9) | | 45 (33.1) | 14 (31.8) | |
| **US findings** | | | | | | |
| **BI-RADS** | | | 0.526 | | | 0.578 |
| 4A | 52 (15.2) | 13 (12.6) | | 18 (13.2) | 5 (11.4) | |
| 4B | 57 (16.7) | 17 (16.5) | | 23 (16.9) | 12 (27.3) | |
| 4C | 84 (24.6) | 28 (27.2) | | 42 (30.9) | 10 (22.7) | |
| 5 | 62 (18.2) | 25 (24.3) | | 25 (18.4) | 7 (15.9) | |
| 6 | 86 (25.2) | 20 (19.4) | | 28 (20.6) | 10 (22.7) | |
| **Multifocality** | | | 0.243 | | | 0.217 |
| yes | 78 (22.9) | 18 (17.5) | | 15 (11.0) | 8 (18.2) | |
| no | 263 (77.1) | 85 (82.5) | | 121 (89.0) | 36 (81.8) | |
| **Maximum diameter (cm)** | | | **<0.001** | | | 0.060 |
| <2 | 268 (78.6) | 57 (55.3) | | 67 (49.3) | 14 (31.8) | |
| 2-5 | 73 (21.4) | 30 (29.1) | | 66 (48.5) | 30 (68.2) | |
| >5 | 0 (0.0) | 16 (15.5) | | 3 (2.2) | 0 (0.0) | |
| **Tumor shape** | | | 0.729 | | | 0.117 |
| regular | 20 (5.9) | 7 (6.8) | | 13 (9.6) | 1 (2.3) | |
| unregular | 321 (94.1) | 96 (93.2) | | 123 (90.4) | 43 (97.7) | |
| **Margin** | | | 0.219 | | | 0.912 |

| | | | | | | |
|---|---|---|---|---|---|---|
| distinct | 32 (9.4) | 14 (13.6) | | 39 (28.7) | 13 (29.5) | |
| Indistinct | 309 (90.6) | 89 (86.4) | | 97 (71.3) | 31 (70.5) | |
| **Inner echo** | | | 0.082 | | | 0.841 |
| even | 61 (17.9) | 11 (10.7) | | 17 (12.5) | 5 (11.4) | |
| uneven | 280 (82.1) | 92 (89.3) | | 119 (87.5) | 39 (88.6) | |
| **Calcification** | | | **<0.001** | | | **<0.001** |
| present | 123 (36.1) | 86 (83.5) | | 16 (11.8) | 36 (81.8) | |
| absent | 218 (63.9) | 17 (16.5) | | 120 (88.2) | 8 (18.2) | |
| **Color Doppler flow** | | | 0.152 | | | 0.979 |
| rich | 323 (94.7) | 101 (98.1) | | 3 (2.2) | 1 (2.3) | |
| poor | 18 (5.3) | 2 (1.9) | | 133 (97.8) | 43 (97.7) | |
| **Aspect-ratio** | | | **<0.001** | | | **<0.001** |
| ≥1 | 10 (2.9) | 24 (23.3) | | 33 (24.3) | 33 (75.0) | |
| <1 | 331 (97.1) | 79 (76.7) | | 103 (75.7) | 11 (25.0) | |

Abbreviations: SLN, sentinel lymph node; OUQ, outer upper quadrant; OLQ, outer lower quadrant; IUQ, inner upper quadrant; ILQ, inner lower quadrant; HR, hormone receptor; TNBC, triple-negative breast cancer; ER, estrogen receptor; PR, progesterone receptor; US, ultrasound.

Bold value indicates statistical significance.

**TABLE S2 |The comparison of the three researches**

| Research | SOUND | NAUTILUS | Nomogram |
|---|---|---|---|
| **Study type** | Clinical trial (international) | Clinical trial (international) | Retrospective study (2 centers) |
| **Condition /disease** | Early-stage BC (<2cm) | Clinical T1, T2 BC (<5cm) | BC (any size) |
| **Intervention/treatment** | SLNB vs Observation | BCS + SLNB(-) vs BCS +SLNB | BCS + SLNB +ALND(-/+) |
| **Outcome measures** | dDFS, DFS, OS,<br>cumulative incidence of distant recurrences,<br>cumulative incidence of axillary recurrences | iDFS, OS, DMFS,<br>axillary recurrence rate,<br>LRR, AEs,<br>evaluate quality of life | SLN metastasis or not |

Abbreviations: BC, breast cancer; SLNB, sentinel lymph node biopsy; dDFS, distant-disease free survival; DFS, disease free survival; OS, overall survival; BCS, breast conserving surgery; iDFS, invasive disease-free survival; DMFS, distant metastasis free survival; LRR, locoregional recurrence rate; AEs, adverse events; ALND, axillary lymph node dissection.
